# Recent Perspectives on Genome, Transmission, Clinical Manifestation, Diagnosis, Therapeutic Strategies, Vaccine Developments, and Challenges of Zika Virus Research

**DOI:** 10.3389/fmicb.2017.01761

**Published:** 2017-09-14

**Authors:** Apoorva Shankar, Amulya A. Patil, Sinosh Skariyachan

**Affiliations:** ^1^R&D Centre, Department of Biotechnology Engineering, Dayananda Sagar Institutions Bengaluru, India; ^2^Visvesvaraya Technological University Belagavi, India

**Keywords:** mosquito-borne flavivirus, Zika virus (ZIKV), current animal models, vaccine development, computational drug discovery, novel molecular targets, ZIKV research

## Abstract

One of the potential threats to public health microbiology in 21st century is the increased mortality rate caused by Zika virus (ZIKV), a mosquito-borne flavivirus. The severity of ZIKV infection urged World Health Organization (WHO) to declare this virus as a global concern. The limited knowledge on the structure, virulent factors, and replication mechanism of the virus posed as hindrance for vaccine development. Several vector and non-vector-borne mode of transmission are observed for spreading the disease. The similarities of the virus with other flaviviruses such as dengue and West Nile virus are worrisome; hence, there is high scope to undertake ZIKV research that probably provide insight for novel therapeutic intervention. Thus, this review focuses on the recent aspect of ZIKV research which includes the outbreak, genome structure, multiplication and propagation of the virus, current animal models, clinical manifestations, available treatment options (probable vaccines and therapeutics), and the recent advancements in computational drug discovery pipelines, challenges and limitation to undertake ZIKV research. The review suggests that the infection due to ZIKV became one of the universal concerns and an interdisciplinary environment of *in vitro* cellular assays, genomics, proteomics, and computational biology approaches probably contribute insights for screening of novel molecular targets for drug design. The review tried to provide cutting edge knowledge in ZIKV research with future insights required for the development of novel therapeutic remedies to curtail ZIKV infection.

## Introduction

Zika virus (ZIKV) is a mosquito-borne flavivirus (family Flaviviridae) pertinent to West Nile virus, dengue virus (DENV), and yellow fever virus. ZIKV carry a positive RNA which is single stranded (Abushouk et al., 2016). ZIKV has been isolated on many instances from *Aedes africanus* although there was no indication to cause human disease (Petersen et al., 2016). The ZIKV was mostly restricted to African continent and later it was reported in South East Asia (1980) (Saiz et al., 2016). An outbreak of ZIKV divulged in Yap Island, Federated States of Micronesia in 2007. Prior to 2007, 14 cases of human infections were reported (Avsic Zupanc and Petrovec, 2016). An outburst occluded in French Polynesia in 2013 along with dengue pandemic and during this outburst, primitive malformations such as Guillain–Barre syndrome (GBS) and microcephaly were noticed in patients (Musso and Gubler, 2016). The areas infected by ZIKV as per the current knowledge is shown in **Table 1**.

**Table 1:** Table depicting ZIKV cases reported as of August 2017 and category classification by WHO (European Centre for Disease Prevention and Control [ECDC], 2017).

Country	Region	Classification category of country for ZIKV transmission as per WHO	Number of cases reported
American Samoa	American Samoa	Category 3 (interrupted transmission areas)	51
Angola	Angola	Category 1 (virus transmission areas followed by new/re introduction of virus)	2
Argentina	Chaco, Formosa, Salta, Tucuman	Category 1 (virus transmission areas followed by new/re introduction of virus)	4
Bahamas	Bahamas	Category 1 (virus transmission areas followed by new/re introduction of virus)	25
Bangladesh	Bangladesh	Category 2 (virus transmission areas following previous circulation of virus)	1
Brazil	Acre, Alagoas, Amapa Amazonas, Ceara, Distrito Federal, Espirito Santo, Goias, Mato Grosso, Mato Grosso Do Sul, Minas Gerais, Para, Paraiba, Parana, Pernambuco, Piaui, Rio Grande Do Norte, Rio Grande Do Sul, Rondonia, Roraima, Santa Catarina, Sao Paulo, Sergipe, Tocantin	Category 1 (virus transmission areas followed by new/re introduction of virus)	27
	Bahia, Maranhao	Category 2 (newly documented intense transmission areas)	
	Rio de Janeiro	Category 2 (virus transmission areas following previous circulation of virus)	
Colombia	Colombia	Category 1 (virus transmission areas followed by new/re introduction of virus)	46
Costa Rica	Costa Rica	Category 1 (virus transmission areas followed by new/re introduction of virus)	23
Dominican Republic	Dominican Republic	Category 1 (virus transmission areas followed by new/re introduction of virus)	30
India	Gujarat, Tamil Nadu	Category 2 (virus transmission areas following previous circulation of virus)	2
Indonesia	Bali, Bangka Belitung, Banten, Bengkulu, Daerah Istimewa Yogyakarta, Dki Jakarta, Gorontalo, Jambi, Jawa Barat, Jawa Tengah, Jawa Timur, Kalimantan Barat, Kalimantan Selatan, Kalimantan Tengah, Kalimantan Timur, Kepulauan-Riau, Lampung, Maluku, Maluku Utara, Nanggroe Aceh Darussalam, Nusa Tenggara Barat, Nusa Tenggara Timur, Papua, Papua Barat, Riau, Sulawesi Barat, Sulawesi Selatan, Sulawesi Tengah, Sulawesi Tenggara, Sulawesi Utara, Sumatera Barat, Sumatera Selatan, Sumatera Utara	Category 2 (virus transmission areas following previous circulation of virus)	31
Malaysia	Malaysia	Category 2 (virus transmission areas following previous circulation of virus)	8
Mexico	Aguascalientes, Baja California, Baja California Sur, Campeche, Chiapas, Coahuila, Colima, Guerrero, Hidalgo, Jalisco, Michoacan, Morelos, Nayarit, Nuevo Leon, Oaxaca, Puebla, Quintana Roo, San Luis Potosi, Sinaloa, Sonora, Tabasco, Tamaulipas, Veracruz, Yucatan, Zacatecas	Category 1 (virus transmission areas followed by new/re introduction of virus)	25
Philippines	Philippines	Category 2 (virus transmission areas following previous circulation of virus)	57
Thailand	Thailand	Category 2 (newly documented intense transmission areas)	33
United States of America	Cameron	Category 1 (virus transmission areas followed by new/re introduction of virus)	202
	Broward, Miami-Dade, Palm Beach, Pinellas	Category 3 (interrupted transmission areas)	
Vietnam	Vietnam	Category 2 (newly documented intense transmission areas)	232

The ZIKV infection accreted severe global health issues due to the rise in number of GBS correlated reports and exuberant microcephaly cases in infants and fetus in Brazil by the end of 2015 (Saiz et al., 2016). The serosurveys determined wider geographic distribution which included East Africa, Malaysia, India, Nigeria, Thailand, Egypt, Philippines, and Vietnam (Petersen et al., 2016).

More than 5600 microcephaly cases in neonates have been well-documented which delineate greater than 20-fold with respect to historical average of past 5 years by 2016. The mortality rate due to microcephaly associated with ZIKV was registered to be approximately 120 by Brazilian health authorities (Noronha et al., 2016).

## Outbreaks

According to the recent report, there were active circulations of ZIKV nearly all Caribbean and Latin American countries (World Health Organization [WHO], 2017b). Even though the transmission of virus was reported 60 years ago in Africa, the awareness of ZIKV to cause potential threats was revealed post-Brazilian and French Polynesian outbreaks (Baud et al., 2017). The circulation of the virus was recently reported in Southeast Asia and Africa, however; the incidence in these areas was undefined (World Health Organization [WHO], 2017b). The detection of infection in travelers was the basis for ZIKV circulation in Maldives or Vanuatu (Musso et al., 2016). Further, ZIKV has been caused minor outbreaks in Florida and Texas, United States. Recent advances in the diagnostic practices and surveillance system provides examination and reporting of autochthonous cases (Baud et al., 2017). Recent reports revealed that three cases of ZIKV infections in Ahmedabad, Gujarat, India by Ministry of Health and Family Welfare, Government of India (World Health Organization [WHO], 2017a). The cases of ZIKV in India was confirmed through reverse transcription polymerase chain reaction (RT-PCR) by routine laboratory surveillance (World Health Organization [WHO], 2017b). It is estimated that most of the tropical and subtropical areas in the world are at risk for ZIKV, yellow fever virus, chikungunya, and DENV. Studies suggested that the transmissions of ZIKV are evident in 84 countries across the world since April 2007 to March 2017 (Baud et al., 2017).

## Genome

**FIGURE 1:**
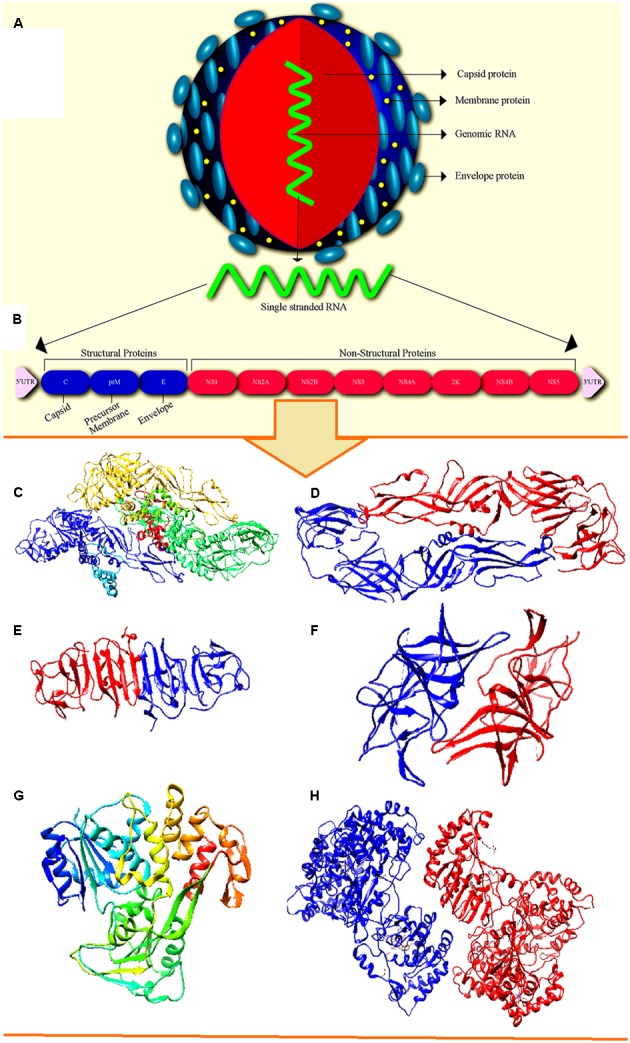
Structure of Zika virus (ZIKV). **(A)** Ultra structure of ZIKV with major surface proteins. **(B)** Genomic RNA of ZIKV exhibiting major structural and non-structural proteins. The crystal structure of available structural and non-structural protein of ZIKV. **(C)** The cryo-EM structure of ZIKV (PDB: 5IRE). **(D)** Structure of envelop protein (PDB: 5JHM). **(E)** Structure of NS1 protein (PDB: 5IY3). **(F)** Structure of the NS2b–NS3 complex (PDB: 5GXJ). **(G)** Structure of NS3 helicase (PDB: 5JPS). **(H)** Structure of NS5 protein (PDB: 5U0B).

ZIKV virion nucleocapsid exhibits icosahedral symmetry which is approximately 50–60 nm in size as shown in **Figure 1A** (Dasti, 2016). The genome is 10,794 kb in length which consists of single-stranded, positive sense RNA, flanked by two non-coding regions (5′ and 3′ NCR) and single open reading frame (ORF) coding for a polyprotein 5′-C-prM-E-NS1-NS2A-NS2B-NS3-NS4A-NS4B-NS5-3′. It is further cleaved into three structural proteins such as capsid (C), envelope (E), membrane precursor (prM) and seven non-structural (NS) proteins which are essential for the replication and assembly of the virus (Faye et al., 2014; Hamel et al., 2016; Rather et al., 2017). The major structural and NS proteins present in ZIKV genome is shown in **Figure 1B**. The three-dimensional structures of gene products of major structural and NS proteins are available till date are illustrated in **Figures 1C–H**. The virus initially infected in wild primates causing erratic “spillover” infections in human when the full-length of ZIKV genome was announced in 2007. It is assumed that after the loss of NS1 codon from the genome, the virus recently adapted to humans (Abushouk et al., 2016). In a study conducted in 2014 with 37 isolates showed that the virus has attained molecular changes to become accustomed to *Aedes dalzieli* by losing a glycosylation site (N154) in their protein envelope (Faye et al., 2014).

The ORF codes a polyprotein of approximately 3400 amino acids which is predicted to be cleaved into mature viral proteins (Saiz et al., 2016). The 5′ UTR which is 106 nucleotides in size displayed conserved folding pattern in mosquito-borne flavivirus (Ye et al., 2016). The genome lacks poly-A tail at the 3′ end and stops in conserved 5′-CU-3′. The G + C content ranges from 50.94 to 51.26%. The sHP-3′ SL structure at 3′-UTR and the Y-shaped stem loop (SLA) structure at 5′-UTR in the flavivirus genome are among the most conserved secondary RNA structures, which is also expected to present in ZIKV. The conserved short sequences at 3′ terminal consists of 5′-ACAG-3′ and 5′-CU-3′ in the top loop of the smallest sHP-3′ SL structure (Zhu et al., 2016).

The lipid bilayer (surrounded by 180 units of glycoprotein E and M) involved in the binding of cell receptors form the envelope (Silva and Souza, 2016). E protein (approximately 53 kDa) is the chief surface protein which functions in diverse aspects of binding, membrane fusion and replication cycle (Faye et al., 2014). In flavivirus genome, *cis* acting RNA elements plays crucial role in viral replication, translation, and pathogenesis (Ye et al., 2016).

The two known ZIKV lineages belong to African lineage (divided into two clades signifying two different introductions) and Asian lineage are differentiated by complete genetic analysis of RNA sequences (Chang et al., 2016). A strong conservation has been observed at nucleotide level among all ZIKV strains with less than 12% divergence. The existing strain belongs to Asian subtype consisting of more than 99.7% of nucleotides and 99.9% of sequence identity with French Polynesian strain of 2013 outbreak as per the phylogenetic analysis (Enfissi et al., 2016). The conservations present in ZIKV strains are essential for diagnostic assays that depend on specific sequence and epitopes (Petersen et al., 2016).

The region of glycoprotein E which is contiguous to Asn 154 glycan depicts the largest structural deviation from other flavivirus. Novel anti-ZIKV vaccines and drugs can be developed based on the knowledge of the function of glycans and proteins (Boeuf et al., 2016).

## Viral Multiplication and Propagation

**FIGURE 2:**
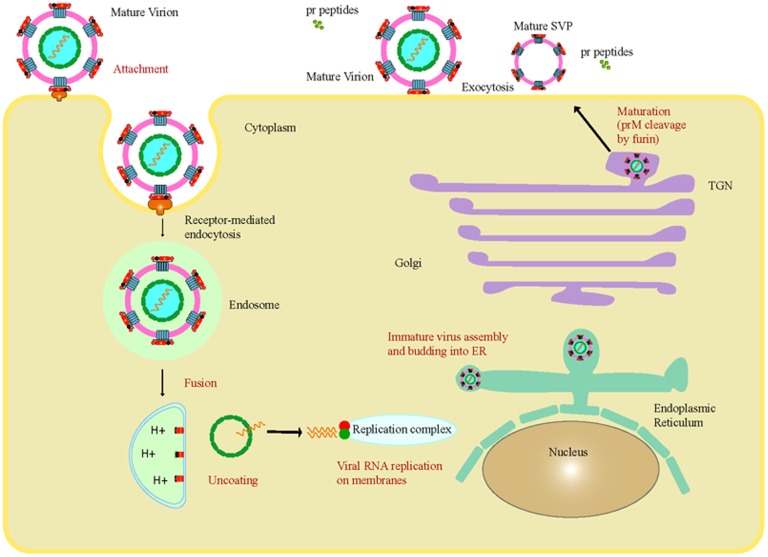
The major events occurred in the replication and maturation of ZIKV inside the host cell followed by their release into the environment resulted in spread of the disease.

The combination of viral envelope with endosome membrane from the host cell set off the mechanism of penetration of flavivirus genome into the cytoplasm by a method triggered by acidic pH inside the cellular endosomes (Saiz et al., 2016). The host cell membrane of flavivirus is known to produce suitable environment for viral replication in endoplasmic reticulum (ER). The major events occurred in the replication and maturation of ZIKV inside the host cell followed by their release into the environment causing spread of the infection is illustrated in **Figure 2**. Most of the arboviruses are known to replicate within the skin dendrites at the primary inoculation site, later, it spreads to the regional lymph nodes followed by the blood stream (Abushouk et al., 2016).

The replication cycle is completed in four stages, i.e., translation of RNA into viral proteins, replication of viral RNA (vRNA), assembly of viral particle in ER and release of the virions (Hamel et al., 2016). The replicates of ZIKV were found in the salivary gland and mid-gut of *Aedes* mosquitoes and *in vitro* cultured mosquito cells C6/36 when infected by blood meal experimentally (Li et al., 2012). The viral infectivity and replication is enhanced by salivary gland products of the mosquito (Malone et al., 2016).

There are various relations with cellular organelles may exist to assist the viral replication, evasion, and propagation in the cellular cytoplasm, even though the exact process needed to be studied (Hamel et al., 2016). It has been suggested in infected mice that the virus replicates mainly in the brain cells, nervous and astroglial cells, whereas, it can replicate *in vitro* in cultured monkey cell lines such as LLC-MK2 or Vero (Saiz et al., 2016).

Defective neurogenesis and anomalous activation of autophagy was caused by the inhibition of Akt-mTOR pathway due to ZIKV infection of human fetal neural stem cells (fNSCs). Autophagy developed as vital ancient immune response during evolution of lysosome-mediated catabolic process (Liang et al., 2016). Some viruses such as flaviviruses took over the cellular autophagy pathways to benefit their life cycle even though the host have progressed autophagy to maintain cellular homeostasis and circumvent viral infection (Heaton and Randall, 2011). Autophagy is induced in multiple cell types including fNSCs due to ZIKV infection (Liang et al., 2016). The various stages present in ZIKV replication are obscure, nevertheless, they are assumed to be similar to other members of flavivirus (Hamel et al., 2016).

## Transmission

The transmission of the diseases through mosquito bite is a common scenario in ZIKV infection. The existence of ZIKV in the pharynx and saliva of infected patients could be persistent with probable transmission. Following the primary isolation of *Aedes aegypti* (other than *A. africanus*) from a group of mosquitoes garnered in Malaysia (1966), it furnished the indication of the first transmission cycle of ZIKV in urban areas (Saiz et al., 2016).

Mosquito bites are the major mode of transmission; however, few cases of non-vector-borne infections have been reported, known as perinatal transmission (Hamel et al., 2016; Rather et al., 2017). The probable modes of perinatal transmission are transplacental, during delivery and at the time of breastfeeding (Besnard et al., 2014).

The occurrence of viable ZIKV particles in blood bags might have severe ramification in pregnant women (Silva and Souza, 2016). Various non-vector modes of ZIKV transmission are congenital and sexual. Separation of the virus or anti-ZIKV antibodies was indicated in various animal reservoirs from domestic and wild animals and numerous non-human primates (Plourde and Bloch, 2016).

The existence of ZIKV nucleic acids were authenticated among 2.8% of asymptomatic blood donors and transmitted infection by transfusion was observed in French Polynesia. Viable ZIKV particles have been isolated from the saliva and urine collected from two acute infected patients, hence; they were recognized as vectors for the transmission (Silva and Souza, 2016). The identification of ZIKV RNA and proteins in conserved microcephalic fetus of Brazilian women at the eighth week of gestation provided the first acceptable proof of vertical transmission (Shastry et al., 2016). The other vectors for ZIKV are *Aedes furcifer*, *A. vittatus*, *A. dalzieli*, *A. metallicus*, *A. hirsutus*, *A. unilinaetus*, *A. africanus*, *A. taylori*, *A. hensillican*, and *A. luteocephalus* (Dasti, 2016). Various species of mosquito such as *A. albopictus* and *A. aegypti* causes difficulties in control and transmission of ZIKV (Saiz et al., 2016). These mosquitoes are also broadening the multiplication of dengue and chikungunya virus (Centers for Disease Control and Prevention [CDC], 2016).

## Studies in Animal Models

### Subcutaneous Route

The crucial requirement to understand the features of ZIKV infection paves path for enhanced research on animal models. In a study conducted by Kumar et al. (2017), immunocompetent guinea pigs sensitive to PRVABC59 (American) strains of ZIKV was utilized. A total of 10^6^ plaque-forming units of the virus were inoculated into the guinea pigs (Dunkin-Hartley) by virtue of the subcutaneous route leading to the recognition of clinical features. Quantitative real-time polymerase chain reaction (qRT-PCR) was employed to determine the viral load in the tissues, protein levels of cytokine, anti-ZIKV neutralizing antibody, and viremia followed by plaque reduction neutralization test (PRNT) and multiplex immunoassay. The symptoms observed were lethargy, hunched back, fever, reduced mobility, and rumpled fur. PRNT presented the anti-ZIKV neutralizing antibody in the infected guinea pigs and the infection led to rise in multiple cytokines level and increased level of growth factors in the serum. The virus was observed to be replicated in the brain and spleen (Kumar et al., 2017).

The response of T-cells and innate immunity to ZIKV has been studied in mouse models. The production of α, β (type I), γ (type II), and aaa (type III), interferon (IFN) and copious IFN-stimulated genes were stimulated by ZIKV that prevented the infection (Bayer et al., 2016; Hamel et al., 2016; Quicke et al., 2016). The mechanism of immune evasion was species specific which contributed to the lack of ability of ZIKV to replicate vigorously which is the root cause of disease in immunocompetent mice (Mysorekar and Diamond, 2016). A defensive role for CD8^+^ T cells was defined in the studies of mouse models of numerous flavivirus. It was observed that animals lacking MHC class I or CD8 possess condensed capacity for viral clearance and adoptively transferred cells might be protective. Subsequent to flavivirus infection, CD8^+^ T cell responses were easily detectable and both virus type specific and cross reactive determinants were targeted (Pierson and Graham, 2016).

The infection caused by Asian-lineage of ZIKV is affiliated with fetal abnormalities. However, poor knowledge of the mechanism has led to the study of rhesus macaque animal model. The study exhibited the injection of vRNA in the plasma approximately 10 days. The infection evoked an immune response such as neutralizing Ab that enable protection in the case of further infection and T-cell response specific for the virus. Nonetheless, the vRNA was detected in saliva and urine after being cleared from blood and further, the presence in CSF indicated that probable existence of ZIKV in some tissues at low levels (Dudley et al., 2016).

A number of clinical studies have examined malformations in the eye and pathology of newborns to mothers infected with ZIKV during pregnancy. Eye diseases in neonates with ZIKV included optic neuritis, colobomas, bilateral iris, chorioretinal atrophy, intraretinal hemorrhages, blindness, and lens subluxation. Uveitis is a viral infection due to the inflammation of uveal tissues (choroid, iris, retina, and ciliary body) which can resulted in the permanent loss of vision, if untreated (Miner et al., 2016). The anterior chamber of the eye enclosed vRNA from the sample fluid signifying the replication of ZIKV within the eye at a certain phase of their clinical syndrome (Furtado et al., 2016). Even weeks after the resolution of viremia and clinical symptoms, ZIKV infection to the eye and testis in the human and animal models were provided information that immune privileged organs might influence the replication (Lazear et al., 2016). Direct targeting of the cells in fetal eyes might cause congenital ocular infection by ZIKV. On the other hand, neurodevelopmental defect which is observed in microcephaly cases is due to non-existence of an infectious cause (Miner et al., 2016).

### Intra-vaginal Route

**FIGURE 3:**
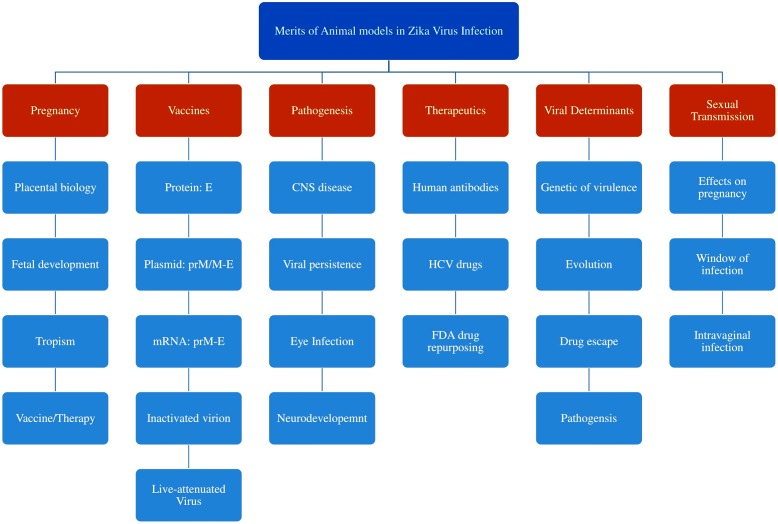
Various applications of animal models in ZIKV research included the study of pathogenesis and transmission, viral determinants and developments of vaccines and therapeutics.

Recent study revealed that the ability of ZIKV vectors to transmit via sexual routes. The study demonstrated by the introduction of the virus into AG129 mice and LysMCre^+^ IFNAR^fl/fl^ C57BL/6 via vaginal route post-hormonal treatment. AG129 mice failed to persist infection in di-estrus-like phase while, infection during estrus-like phase demonstrated resistance. However, LysMCre^+^ IFNAR^fl/fl^ mice in di-estrus-like phase showed short-term infection. The viral replication occurred in brain and spleen along with viremia in vaginal washes 10 days of post-infection. Thus, the study indicated that the occurrence of transgenital transmission accompanied by variation in hormones of female reproductive tract with prolonged prevalence of viral replication (Tang et al., 2016). The applications of studies in various animal models in Zika viral research is shown in **Figure 3**.

### Clinical Manifestations

#### Signs and Symptoms

ZIKV infections in most cases are alleged to be asymptomatic or faintly symptomatic. Most of the generally described symptoms include fever, arthralgia, rash, fatigue, myalgia, conjunctivitis, and headache. The growth stage from bite of mosquito to outbreak of the symptom is approximately 3–12 days in humans (Plourde and Bloch, 2016). After which a mild fever with wide range of symptoms are appeared for 2–7 days. The outburst of maculopapular rashes shown by more than 90% of patients is the major clinical symptom that describes ZIKV infection (Hamel et al., 2016). Typically, whole blood count is normal even if the count changes inconsistently non-specific (mild neutropenia, mild lymphopenia, and mild to moderate thrombocytopenia) (Campos et al., 2015). An inherent incubation period of 4–5 days within the human host later, infecting another vector during blood feeding where it spend an extrinsic incubation period of 8–12 days and propagates via saliva of the vector to another host (Abushouk et al., 2016). Only approximately 18% cases of ZIKV infections were recorded to be symptomatic, where it causes self-limiting, mild disease with up to 10 days of incubation period, normally flawed with other arboviral infections such as chikungunya or dengue (Saiz et al., 2016).

GBS includes the damage of peripheral nervous system with loss of myelin insulation in facial palsy, muscle dysfunction and myalgia. Fetal head circumference below average for gestational age with majority resulting in disabilities such as physical disability and intellectual retardation is defined as congenital microcephaly (Hamel et al., 2016). The prevalence of GBS or microcephaly presented to be analogous between symptomatic and asymptomatic cases (Koenig et al., 2016). The current outbreak in Brazil provided prodigious attention to microcephaly cases mostly due to the significant increase in infant microcephaly cases (Dasti, 2016).

The noteworthy cellular death of ZIKV infected neural stem cells provided by the latest studies open the role of inhibition on fetal brain development by ZIKV (Tian et al., 2016). A study revealing the viral neutropism in mice that were infected intraperitoneally and development of the disease in mouse brains that were directly infected provide credible neuropathological relation between ZIKV and CNS anomalies. In contradiction to dengue, fatality from acute ZIKV infection is limited; however, it has been reported in children with sickle cell disease in Colombia. In ZIKV infected patients, hemorrhagic signs have not been reported (Koenig et al., 2016).

There is limited knowledge on the presence of ZIKV in congenitally infected newborns but the virus is observed to be linked with microcephaly. There was a failure in the detection of ZIKV during the initial physical examination when the infected mother (26 weeks of pregnancy) experienced symptoms and gave birth to a microcephaly infected male child in 2016 in Sao Paulo, Brazil. The weight, length, and head circumference of the infant was observed to be 3095 g, 48 cm, and 32.5 cm, respectively. A reduced brain parenchyma, preferentially in the frontal and parietal lobes, foci of calcification in the sub-cortical area and compensatory dilatation of the infratentorial supraventricular system was observed by magnetic resonance imaging technique. The saliva and urine were tested for the virus on the 54th day by qRT-PCR. The studies depicted that all the three assays were positive for ZIKV RNA with 1.4 × 10^5^, 4.1 × 10^4^, and 5.4 × 10^3^ copies per milliliter in the serum, saliva, and urine, respectively. A high degree of similarity was observed in the infant with bootstrap value of 98.5% when RNA sequencing of urine samples were performed along with positive results for IgM and IgG. The detection of ZIKV RNA in the serum continued on day 67 with 2.8 × 10^4^ copies per milliliter by qRT-PCR. On day 216, there was failure to detect ZIKV RNA in the serum and ZIKV-specific IgG titer was elevated (>320) in comparison with the first and second samples (average titer, <99). However, the newborn showed delay in neuropsychomotor development, with spastic hemiplegia and global hypertonia, with severe damage to the right dominant side by the age of 6 months (Oliveira et al., 2016).

### Recent Diagnostic Strategies

The symptoms of ZIKV infection are non-specific and can be confound with symptoms of other diseases such as chikungunya and dengue caused by arbovirus, the differential laboratory diagnosis plays vital role in the regions where the virus co-circulates and hence, ZIKV infection can be misdiagnosed (Zanluca and Dos Santos, 2016). Asymptomatic individuals might be important reservoirs for virus transmission and have obscure diagnostics. All the currently available diagnostics have been used to test symptomatic individual (Boeuf et al., 2016). Date of onset of the illness for ZIKV infection is intricate to ascertain because of sporadic and frequent mild fever (Gourinat et al., 2015).

Laboratory methods analyze ZIKV infection by the detection of viral nucleic acid and viral antibody or antigen (Zanluca and Dos Santos, 2016). During symptomatic period of infection, various laboratory parameters and symptoms provide information on ZIKV infection such as leucopenia, thrombocytopenia, serum lactate dehydrogenase, γ-glutamyl transferase, and elevated protein markers. The commercial assays and PCR-based assays accepted by the Communaute Europeenne and serological assays permitted by US Food and Administration (FDA) can be used in emergency situations (Plourde and Bloch, 2016).

In the earlier stages, mainly during primary acute infections, techniques such as solid phase immunosorbent assay and hemagglutination were used to diagnose flaviviral infection (Chang et al., 2016). The detection of viral nucleic acid was performed by RT-PCR and IgM capture enzyme-linked immunosorbent assay (MAC-ELISA). Even though the exact onset timing and duration of IgM antibody response to the virus can be detected by MAC-ELISA has not been established, clear understanding which implies that IgM become visible as viremia waves within the first week after the onset of symptom and persevere for several months. The Arboviral Diagnostics and Reference Laboratory at CDC developed IgM ELISA to detect the samples obtained from Yap Island outburst (Saiz et al., 2016).

RT-PCR analysis of serum samples attained inside the first week of clinical illness and MAC-ELISA testing of samples that were not tested or found negative by RT-PCR was expected to have the highest diagnostic yield. The uses of RT-PCR were found in detecting ZIKV in amniotic fluid, breast milk, semen, saliva, and blood products. The utility of urine samples for diagnosis of ZIKV infection demonstrated that ZIKV RNA detectable at an elevated load longer period than in serum (Gourinat et al., 2015). The positive results should be confirmed by neutralization assay such as PRNT which offers greater specificity, whereas, some patients with the probability of being infected by another flavivirus exhibit fourfold or higher rise in neutralizing antibody titers against other flavivirus (Zanluca and Dos Santos, 2016). Neutralizing properties of ZIKV antibodies have been subjected to PRNT and played a major role in the diagnosis and case classification (confirmed, probable, suspected, and no ZIKV infection) of several patients during 2007 outbreak on Yap Island (Marano et al., 2016).

In accordance with the recent CDC guidelines for testing pregnant women likely to be exposed to ZIKV, there was a strong explanation for testing asymptomatic pregnant women as long as sufficient laboratory facilities are available (Boeuf et al., 2016). The most specific diagnostic approach is the molecular amplification of serum samples and which is the ideal testing method for ZIKV for the period of acute phase of illness. However, serological testing is not recommended as ZIKV IgM might be undetectable during acute phase (Plourde and Bloch, 2016).

### Limitations

The following are the major demerits to be considered during the diagnosis of ZIKV infections (Charrel et al., 2016; Dasti, 2016; Hamel et al., 2016):

• As a result of identical clinical features with other infections of arbovirus such as chikungunya and dengue, the diagnosis of ZIKV is challenging.
• Due to the failure in differential diagnosis, most often, ZIKV is diagnosed as DENV at the incipient stage.
• The scarcity of sensitive and specific laboratory test makes it complicated to detect the virus in the samples collected from the patients.
• Antibodies of ZIKV infection are often cross-reactive with other flavivirus such as yellow fever, dengue, West Nile Virus, Murray valley encephalitis, Kunjin virus and that which frequently co-circulate with ZIKV, confines the use of serology and hinders the regular performance of the viral culture. The lacks of antigenic detection contribute one of the major challenges in the diagnosis.
• Even if PCR-based testing of saliva, blood, and urine is highly recommended, accessibility of the preferred amount of nucleic acids in various samples possess significant encumbrance.
• The short viremic duration demonstrated an impediment in the detection of significant antibodies.
• To run molecular tests in an outburst situation, the laboratories require acquaintance and experience of quality control and validation, ability to access rapid reagents and other necessities from supply chains, and to augment the high throughput testing approaches.

### Prevention and Control

The presence of *Aedes* mosquito enables ZIKV to invade newer areas and impose worldwide risk as there are no preventive approaches or vaccines for ZIKV. The control measures are depending on the eradication of mosquito vector breeding foci (Wahid et al., 2016; Zanluca and Dos Santos, 2016). Methods that sustain the environment and preserve the effectiveness should be employed for efficient control of ZIKV (Chang et al., 2016). With the advent of high level research, effective and safe ZIKV vaccine probably developed in the near future. An initiative for the development of ZIKV vaccine was started by National Institutes of Health (NIH) in 2015 (Wahid et al., 2016).

### Mosquito Control Measures

Entomological surveillance is the fundamental aspect for any vector control program. Countries at greater risk of *Aedes* introduction require vigilant monitoring at the entry point (World Health Organization [WHO], 2016). The control of *A. aegypti* relies on an integrated approach which included the exclusion of breeding sites of *A. aegypti* mosquito, the application of insecticides and larvicides to eliminate fully developed mosquitoes. In order to battle ZIKV infection, mosquito breeding grounds should be primary measured which is carried out by targeting mosquito breeding through eradication of probable egg laying sites with the aids of insecticide treatment or by drying the wet environments (Plourde and Bloch, 2016). Research is being carried out for strategies to accomplish mosquito elimination. For example, in order to curb the breeding or proliferation of the vector species, a survey is being conducted by WHO on the release of sterile irradiated mosquitoes (Shastry et al., 2016). Prevention and control of ZIKV infections are primarily directed toward evading the mosquito bites accountable for transmission of the disease (Saiz et al., 2016).

### Control Measures for Public

New recommendations were provided by FDA for blood and organ donation as donors are asymptomatic at the time of blood donation. An additional proficient tactic to thwart blood-borne transmission of infectious agent is pathogen reduction technology mainly for unknown pathogens (Marano et al., 2016). As of 2016, for donors who are at risk to ZIKV infection in areas without active ZIKV transmission, the FDA advises that donation should be delayed for 4 weeks. FDA suggested that blood or blood products must be obtained from areas in US without active transmission of ZIKV for those residing in the areas of active ZIKV transmission (Food and Drug Administration [FDA], 2016). Those women who are pregnant and ones trying to conceive should not travel to virus affected areas as suggested by ECDC (European Centre for Disease Prevention and Control) and CDC. It is also advised to use precautionary measures such as condoms, while traveling or residing in areas of active virus circulation (Centers for Disease Control and Prevention [CDC], 2016). A travel alert of level 2 (practice enhanced precautions) has been issued by CDC for the people traveling to regions such as El Salvador, Guatemala, Haiti, Brazil, Mexico, Panama, French Guiana, Paraguay, Suriname, Honduras, Commonwealth of Puerto Rico, and Venezuela which have been affected by active transmission of ZIKV (Shastry et al., 2016).

### Environmental Control Measures

The use of insecticides and exclusion of tiny pools of sluggish water are the major vector control strategies (Basarab et al., 2016). The main aspect considered to combat ZIKV is the knowledge about the breeding grounds of mosquitoes such as stagnant water ponds, unattended furniture, watery polythene bags, old automobile tires, risk factors associated with gardening, and plants containing water. However, for medium–large containers that hold water for domestic uses should be enclosed with tight fitting covers to protect from the introduction of eggs by female mosquitoes (World Health Organization [WHO], 2016). The main recommendations for epidemic areas of travel are as follows:

• Prefer complete covering by cloths.
• Accepted mosquito repellents to be used.
• Reside in air conditioned or screened rooms.
• Bed nets to be used if screened or air conditioned rooms are not available or while sleeping in the open.
• Secure eating habits.

### Bacteria Prevents Transmission

**FIGURE 4:**
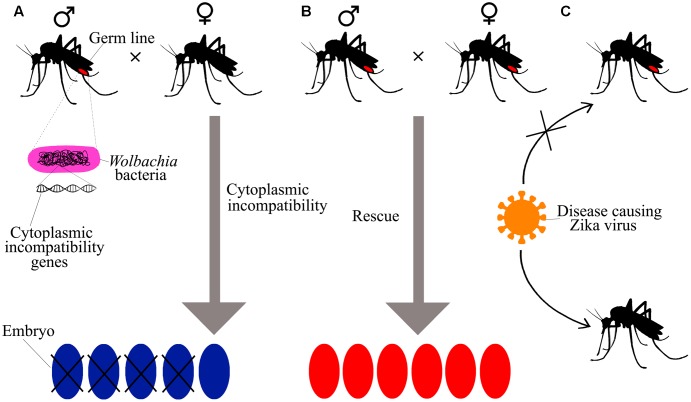
The preventive action of *Wolbachia* spp. on ZIKV by cytoplasmic incompatibility (CI) to prevent transmission of disease. **(A)** The bacteria *Wolbachia* spp. infect the germ lines of mosquitoes and only female can transfer the bacteria to the next generation. The mating between bacteria infected males and uninfected females resulted in embryo lethality are known as CI, a major approach used to restrain insect populations in some pest-management systems. **(B)** CI may prevent in rescue mating between bacteria infected males and bacteria infected females. Because the effect of bacterial infection on the reproduction of insect supports the survival of bacteria infected females over uninfected females, bacteria might quickly spread during population of insect. **(C)** The bacterial infection can avert mosquitoes from being infected by some viruses that infect human host and the spread of the bacteria by an insect population is being used to prevent insect-mediated human diseases as per Sullivan and O’Neill (2017).

The intracellular bacterium *Wolbachia pipientis* reported to be capable candidate for arbovirus control and prevention. *Wolbachia* is found in various species of insect which included butterflies, ants, mosquitoes, bees, and beetles wherein they show endosymbiotic influence on the host replication (Aliota et al., 2016). Cytoplasmic incompatibility (CI) is the usual type of manipulation that permits rapid population dissemination. CI maintains high frequency by patterns of crossing sterile individuals that can provide reproductive benefit to the females carrying bacteria (Molloy et al., 2016). In *A. aegypti*, *W. pipientis* strain wMel applied in field testing that attained at high population frequency (Hoffmann et al., 2014). Since *A. aegypti* has no native *Wolbachia* symbionts, the wMel strains of the bacteria were incorporated into *A. aegypti* which condenses the lifespan of female mosquitoes (Aliota et al., 2016). The proposed mechanism of prevention of ZIKV by *W. pipientis* is shown in **Figure 4**.

The upregulation of innate immune genes has been observed to be added to the phenotype; however, it is not necessary for blockage of viral transmission. Further, the mechanism behind the pathogen inhibition remains uncertain. Since the infected mosquito exhibit decreased fitness in small-scale field releases, *W. pipientis* is not regarded as a biocontrol agent (Nguyen et al., 2015).

## Recent Advancements

### Vaccines

Various vaccines are being developed recently such as recombinant protein subunit and DNA-based vaccine, an inactivated ZIKV vaccine. Subunit vaccines are required in multiple doses to stimulate an immune response although they are safe and can be developed in shorter timeline (Shan et al., 2016). In India, Bharat Biotech, a premier Biotech industry, is dynamically developing two vaccine candidates, i.e., recombinant vaccine and inactivated vaccine which destruct the ability to replicate, whereas the immune system can still detect it. These two candidates are undergoing pre-clinical trials. Recent studies revealed that ZIKV was found to be sensitive to the antiviral effects of type 1 and type 2 IFNs (Fellner, 2016). Anti-viral nucleoside analogs GS-5734 and BCX4430 are currently in the first and second phases of clinical trials (Mumtaz et al., 2016). Further, recent studies showed that ZIKV infection could act in response to IFN therapy (Hamel et al., 2015).

Inactivated Zika vaccine in the purified form was recently developed by Walter Reed Army Institute of Research (WRAIR) where four phase 1 trials have been initiated. A joint collaboration between National Institute of Allergy and Infectious Diseases (NIAID) and University of Pennsylvania, Moderna/Valera and GlaxoSmithKline (GSK) investigated several mRNA vaccines that are identical to DNA vaccines. The candidate developed by Moderna/Valera has been assessed in phase 1 clinical trial (National Institute of Allergy and Infectious Diseases [NIAID], 2017).

AGS-v is a vaccine considered to be acted against several mosquito-borne infections. It is found to set off an immune response to the proteins present in mosquito saliva instead of specific virus. Four synthetic proteins found in mosquito salivary glands are utilized to formulate the test vaccine. The proteins are intended to trigger modified allergic responses that lead to the prevention of infection on being bitten by the mosquito. This vaccine currently evaluated for phase 1 clinical trial at NIH Clinical Center in Bethesda, MD, United States (National Institute of Allergy and Infectious Diseases [NIAID], 2017).

### Therapy and Treatment

An accurate understanding of ZIKV biology is essential for the development of anti-ZIKV therapeutics, vaccines, and improved diagnostics (Boeuf et al., 2016). The treatment for ZIKV infection is completely supportive as no explicit antiviral therapy is established yet (Koenig et al., 2016). Currently, no drugs are permitted for the treatment but numerous nucleoside analogs have some antiviral activity in cell culture such as 2′-C methylated nucleosides like 7-deaza-2′-C methyladenosine (7DMA) and 2′-C methylcytidine (2CMC) and Ribavirin, Favipiravir and T-1105. Initially, 7DMA and 2CMC were developed for the treatment of hepatitis C which is distantly linked to ZIKV. Studies have shown that these compounds have antiviral activities against other flavivirus (Mumtaz et al., 2016). Brazil’s Butantan Institute, Sao Paulo was the first group to announce the development of ZIKV vaccine (Fellner, 2016). To lower the risk of hemorrhage, aspirin and non-steroidal anti-inflammatory drugs should be evaded (Zanluca and Dos Santos, 2016). The administration of fluids and Acetaminophen (Paracetamol) or Dipyrone is suggested to manage fever and pain. The incidence of Reye’s syndrome can be prevented in children below 10 years by eschew of aspirin, while acetaminophen can be used as an alternative.

An integrated advancement in traditional Chinese medicine and western medicine were used to cure ZIKV infection. Xiyanping was used for the treatment of latest case of ZIKV due to the antiviral properties of this drug (Logan, 2016). Intravenous injection of 250 mg Xiyanping was prescribed daily as an antiviral agent in the infectious secluded wards. Chloramphenicol eye drops were prescribed to relieve conjunctival congestion along with Ibuprofen. Further techniques have been suggested by WHO advisory group to manage ZIKV infections (Fellner, 2016). Some of the future medicine toward ZIKV is shown in **Table 2**.

**Table 2:** The potential drug candidates suggested being ideal toward ZIKV and these probably provide insights in future vaccine developments.

Compounds	Compound source
ChEMBL/PubChem:29	FDA approved antiviral drugs
Quinacrine, pyronaridine Chloroquine and amodiaquine Kinase inhibitors Chlorcyclizine NTCP inhibitors vs HepB	FDA drugs that are not antivirals but have shown antiviral activity
Quinacrine, berberine Amodiaquine Prochlorperazine	FDA approved drugs active *in vitro* or *in vivo* vs dengue virus
H-89, MPP, BIBU 1361 Diverse molecules	Other compounds from HTS screen vs dengue virus, yellow fever, etc.
ChEMBL:90–95	Compounds from ChEMBL datasets
PubChem:96–98	Compounds from PubChem

Epigallocatechin gallate (EGCG), a polyphenol (catechin) found in *Camellia sinensis* (green tea) provides intense antiviral activities against the viruses such as herpes simplex virus (HSV), influenza virus (FLU), hepatitis C virus (HCV), and human immunodeficiency virus (HIV) (Carneiro et al., 2016; Elfiky, 2016). Through direct interaction with lipid envelope, the admission of virus into host cell is inhibited by EGCG causing a consequent obliteration of virus particle. Previous studies were showed that EGCG has low membrane permeability, chemically unstable, and is quickly metabolized by the organism. Recent studies have shown that EGCG is noxious to various cell lines at elevated concentration (Carneiro et al., 2016). Over the short period of time, neutralizing antibody preparations, development and testing of antiviral and medicines designed to block Fc receptor interaction are some of the strategies suggested by medical counter measure to manage ZIKV infections.

### Advances in Computational Biology for Drug Development

**FIGURE 5:**
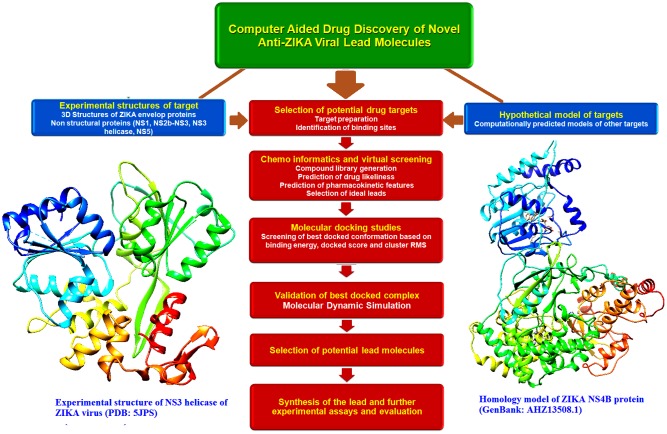
Schematic representation of the major concepts involved in the computational drug discovery of novel anti-Zika viral lead molecules. Drug discovery processes involved in the selection of novel and probable drug targets (both experimental structures and hypothetical models), selection of novel leads with ideal drug likeliness and pharmacokinetic properties, study of target-lead interaction by molecular docking, validation of the best docked poses by molecular dynamic simulation, selection of the best lead molecules, synthesis and large scale screening of the best leads and further experimental evaluation.

A quick development of therapeutic requires the swift analysis of mechanism underlying the pathogenesis of the ZIKV (Basharat et al., 2016). The prM, Env, and NS proteins can be considered as potential drug targets. In the NS3 and NS5 drug-binding pockets, ZIKV shares sequential homology and presumable structural similarities with other flavivirus (Cox et al., 2015). As an early stage of computational protocol, the accessible crystal structures from various parts of the virus and novel homology models were used. However, to process large databases of compound libraries, more sophisticated docking techniques such as Blind Docking simulations could be consequently applied. Furthermore, ligand-based virtual screening (LBVS) methods could be useful for the screening of novel lead candidates. Recent studies have shown that the application of LBVS in discovering potential inhibitors against viruses. The electronic description of biological processes such as molecular dynamics and quantum mechanical calculations, could be applied to further filter the structures that lead the prediction of more accurate binding energies between the receptor and ligand (Cerón-Carrasco et al., 2016). The recent advancement and major concepts (Ramharacka and Soliman, 2016; Munjal et al., 2017) involved in the screening of novel anti-ZIKV lead molecules by computer aided discovery approaches are shown in **Figure 5**.

Recently, small number of FDA-approved drugs such as IFN (34.3 IU/ml), Ribavirin (143 μg/ml), 6-Azauridine (1.5 μg/ml), and Glycyrrhizin (384 μg/ml) were tested against ZIKV (Ekins et al., 2016a,b). The phosphorylation in viral protein assists in replication and interrupts the normal host-cell functions that are responsible for pathogenesis. Additionally, phosphorylation and dephosphorylation of protein residues might be involved in cell signaling. Computational biology tool such as ViralPhos exclusively tailored for phosphorylation analysis of viral proteins and probes the virus substrate motif which aids in the identification of potential phosphorylation sites in the viral protein (Basharat et al., 2016).

To control more than 60% of human protein-coding genes, small non-coding RNAs (sncRNAs) which are termed as MicroRNAs (miRNA) that regulate post-transcriptional gene expression by translational repression were examined (Friedman et al., 2009). The influences of ZIKV/human-host interaction by miRNAs are presented in two hypotheses. Firstly, miRNAs which provide advantages linked with viral and cellular gene expression could be transcribed by the virus (Klase et al., 2013). Secondly, the direct interaction between viral genomes and cellular miRNAs enhanced the potential of viral replication. The human genome miRNAs are found to be possessed few complementary sequences with ZIKV genomes, predicted miRNAs can be targeted to develop anti-ZIKV lead molecules. These miRNAs can be retrieved from ZIKV-CDB database (Pylro et al., 2016). The database is an open-source platform which aids in sharing and identification of potential targets. A valuable knowledge base is provided by the ZIKV-CDB to support researchers to targets the predicted miRNAs. The association between neurobiological development in infants and ZIKV infection can also be steered by experimental investigation. The Genomic and Computational Biology Group continuously curate and maintains the database (Pylro et al., 2016).

Furthermore, there is high scope using alternative approaches other than molecular modeling-based methods. For example, techniques such as advanced machine learning methods (i.e., deep learning) are appropriate to capture complex statistical patterns between thousands of descriptors extracted from drug compounds. Better predictions can be made using Deep Neural Networks (DNN) in comparison with standard machine learning tools based on Kaggle competition data sets. A deep machine learning network was developed to identify the site for epoxidation and to differentiate epoxidized and non-epoxidized molecules (Skalsky and Cullen, 2010; Cerón-Carrasco et al., 2016).

### Challenges for ZIKV Research

The most challenging aspect of ZIKV research is the demonstration of ZIKV-induced *in vivo* congenital alterations. The *in utero* infection on pathology has not been reported in mice, and it is difficult to model this pathology in wild-type pregnant mice. The peripherally inoculation of ZIKV to AG129 pregnant mice causes fetus infection and congenital variations, because flaviviral infection leads to the development of viremia in mice when compared to the ones infected in humans (Rossi et al., 2016).

The nucleoside analogs such as 7DMA and T-705 Favipiravir are proficient for the treatment of several viral infections. However, the application of nucleoside analogs for the treatment of ZIKV can be unsafe in pregnant women. Currently, there are protocols established to assess the safety of Ribavirin treatment during pregnancy. The gestational use of nucleoside analogs is suggested when benefits exceed in comparison to the probable fetal harm. The safety of most nucleoside analogs should be studied for ZIKV-induced congenital malformations to examine whether these drugs exhibited competence in infected adults (Ribeiro et al., 2016).

The preservation of an intact immune response in mice models is essential; however, there are issues with inoculation of the virus to brain. The murine models that exist with sustained viremia are supreme to the compounds such as neutralizing antibodies and antiviral agents, which target ZIKV for preclinical testing. To elucidate the scientific challenges, various mice models of infection are required in ZIKV research (Ribeiro et al., 2016).

An alternative vector strategy to abate the transmission of ZIKV by mosquitoes was demonstrated by the introduction of the bacterium *Wolbachia* into the *A. aegypti* population. Thereby, the replication of ZIKV curtails within the mosquito and delays the distribution of virus to the salivary glands of mosquitoes. The existing limitation for the application of vector control strategies in ZIKV affected areas are the probability of environmental damage and public distrust of genetically modified organisms (Rajah et al., 2016).

## Conclusion

ZIKV infections are spreading across the world and possessed great threat to the public health. The infection often associated with neurological disorders and severe birth defects. Several vector and non-vector-borne forms of transmission are reported which aid in spreading the disease. The similarity of ZIKV with other flavivirus such as DENV and West Nile virus is worrisome. Further, more severe complications such as pathogenesis and cellular mechanism are unknown. Hence, there is a high demand to undertake ZIKV research as thrust areas and develop strategies to ascertain therapeutic remediates to curtail the infection. There are several researchers and industries are working toward the development of effective anti ZIKV vaccines. The development of vaccine requires clear understanding of the pathogenesis and genome structure of the virus. An interdisciplinary environment of *in vitro* cellular assays and computational biology approaches probably contribute insights for the screening of molecular targets in drug design. An improvement in the existing strategies of vector control, awareness programs among people and opening novel outlook in ZIKV research to study the current challenges and limitations are the vital component in the disease management.

## Author Contributions

AS and AP collected the data and prepared the complete manuscript, SS reviewed, edited, and revised the manuscript.

## Conflict of Interest Statement

The authors declare that the research was conducted in the absence of any commercial or financial relationships that could be construed as a potential conflict of interest.
